# Gut microbiome of pre-adolescent children of two ethnicities residing in three distant cities

**DOI:** 10.1038/s41598-019-44369-y

**Published:** 2019-05-24

**Authors:** Wei Wei Thwe Khine, Yuwei Zhang, Gerald Jian Yi Goie, Mung Seong Wong, Mintze Liong, Yeong Yeh Lee, Hong Cao, Yuan-Kun Lee

**Affiliations:** 1 Department of Microbiology & Immunology, Yong Loo Lin School of Medicine, National University of Singapore, 5 Science Drive 2, Singapore, 117545 Singapore; 2 Functional Food Forum, Faculty of Medicine, University of Turku, Turku, 20014 Finland; 3 School of Medical Sciences, Universiti Sains Malaysia, Kubang Kerian 16150, Kota Bharu, Malaysia; 4 Bioprocess Technology, School of Industrial Technology, Universiti Sains Malaysia, Penang, 11800 Malaysia; 5 Department of Microbiology, Public Health School, Southern Medical University, Guangzhou, 510515 P. R. China; 6 Department of Surgery, National University of Hospital, Tower Block, 1E Kent Ridge Road, Singapore, 119228 Singapore

**Keywords:** Metagenomics, Microbiome

## Abstract

Recent studies have realized the link between gut microbiota and human health and diseases. The question of diet, environment or gene is the determining factor for dominant microbiota and microbiota profile has not been fully resolved, for these comparative studies have been performed on populations of different ethnicities and in short-term intervention studies. Here, the Southern Chinese populations are compared, specifically the children of Guangzhou City (China), Penang City (west coast Malaysia) and Kelantan City (east coast Malaysia). These Chinese people have similar ancestry thus it would allow us to delineate the effect of diet and ethnicity on gut microbiota composition. For comparison, the Penang and Kelantan Malay children were also included. The results revealed that differences in microbiota genera within an ethnicity in different cities was due to differences in food type. Sharing the similar diet but different ethnicity in a city or different cities and living environment showed similar gut microbiota. The major gut microbiota (more than 1% total Operational Taxonomy Units, OTUs) of the children population are largely determined by diet but not ethnicity, environment, and lifestyle. Elucidating the link between diet and microbiota would facilitate the development of strategies to improve human health at a younger age.

## Introduction

The gastrointestinal tract contains the greatest number of colonized microbes, mostly anaerobes, estimated to cover 70 percent of all the microbes in the human body^1^. Existing at such high concentrations in our body, it has been found that these gut microbes play important roles in modulating our health and diseases^2–4^. Thus, it is most relevant to understand factors determining gut microbiota profile.

Diet is an important factor determining the dominant gut microbiota^1,5,6^. A western diet consists of high fat, refined carbohydrates, and low fiber, resulting in European having high *Bacteroides* abundance and an underrepresented *Prevotella* genus^7^. Whereas the Africans have high levels of *Prevotella*, resulting from the high fiber content in their diet^7^. This is due to the *Prevotella* genus containing many species with high fiber degrading potential^8^. However, in this and other studies on long-term habitual dietary consumption in distant regions and close vicinity of different ethnicity^9–11^, the contribution of human genes, living environment and lifestyle in the microbiota profile could not be verified.

Many intervention studies had demonstrated that short-term diet changes^12^ and seasonal variation in dietary habit^13^ can affect the gut microbiota, but the magnitude of change did not result in a permanent alteration of enterotype. They also could not verify the factor(s) that determine the stability of the microbiome. A recent publication reported that individuals with distinct ancestral origins who shared a relatively common environment showed significant similarities in the compositions of the microbiomes^14^.

In our project, we focused on three Asian cities, Guangzhou China, Penang West coast Malaysia, and Kelantan East coast Malaysia. The two Malaysian cities are populated by Chinese of Southern China ancestry. All three cities also have the same tropical climate and are all surrounded by sea (food source). Whilst all three population consume Chinese food, Malaysia Chinese also consume the local diet (Malay foods) and local ingredients. For instance, the Peranakans of Penang consume Chinese-Malay fusion food.

This study aims to compare the Chinese (CN) population of Penang (PN), Kelantan (KL), and Guangzhou (GZ) based on their microbiota and diet. Having the same ancestors, it would be interesting to find out if their gut microbiota will be similar due to their race or will it segregate based on their different locations and dietary habits. The proportion of Chinese in the two Malaysian cities is different. The percentage of Malays (ML) and Chinese in Penang are 41.6% and 41.5% respectively, which is approximately equal. As for Kelantan, the percentage of Malays and Chinese in the region is 92.3% and 3.2% respectively, making the Chinese population a minority race in Kelantan^15^. The difference in the proportion of Chinese in the two cities will also result in the difference in the level of influence in the food culture. Hence, the food consumed by the Chinese in Kelantan would have a higher level of influence by the Malay culture than that in Penang. If the hypothesis that diet is the most important influence on gut microbiota, then based on the level of influence in food culture, the gut microbiota of the Penang Chinese should be more similar to that of the Guangzhou Chinese than that of the Kelantan Chinese. The highest difference in gut microbiota should be the highest between that of Guangzhou Chinese and Kelantan Chinese. Besides, Penang is an urban modern city, while Kelantan is rural and largely agricultural. Children were chosen in this study, for they consumed more uniform and traceable home cooked food, and at a critical age whereby the microbiota profile has established to that of the adult-like^16^ and may determine later life health status.

Other than comparing the Chinese population, the Penang and Kelantan Malay samples were also collected for comparison analysis. This is to investigate if the gut microbiota of the Penang and Kelantan Chinese would be similar to that of other Chinese populations from another country (China) or to the Malays who reside in the same city sharing many food types, living environment, and lifestyle.

## Results

### Effect of ethnicity on microbiota

To access the impact of ethnicity on gut microbiota, (1) two different ethnicities; namely, the Malay and Southern Chinese living in Penang and Kelantan, and (2) the Chinese children from Guangzhou, Penang, and Kelantan were compared.

**Figure 1:**
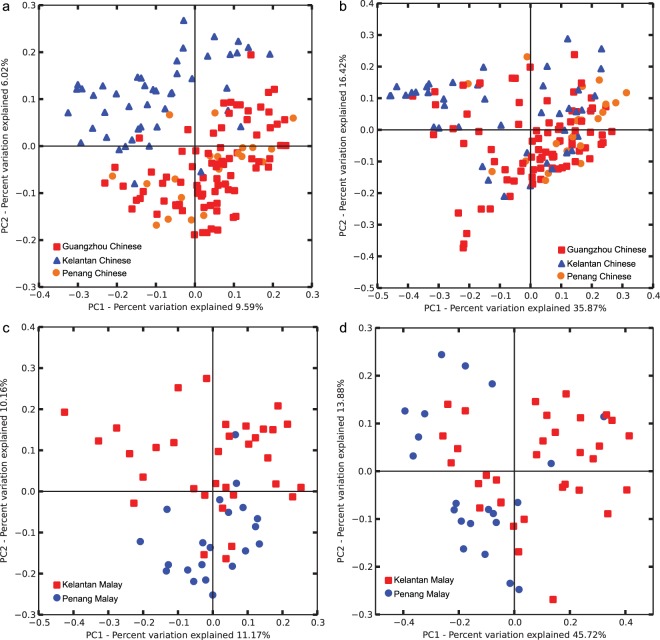
Unweighted and weighted UniFrac Principle Coordinates Analysis (PCoA) plots of microbiota among the same ethnicity. Two different races (Chinese and Malay) of children from Guangzhou, Kelantan, and Penang were compared against each other and visualized in the unweighted (left) and weighted (right) PCoA plots (**a**,**c** and **b**,**d**). The statistical analysis of ANOSIM was shown in Supplementary Table S1.

Firstly, the unweighted Principle Coordinates Analysis (PCoA) plot of the Chinese population showed a separation between the Guangzhou and the Kelantan children (Fig. 1) at a p-value of 0.001 statistically and comparatively high R-value of 0.517 (Supplementary Table S1). There was also a separation between the Chinese children of Guangzhou and Penang (p = 0.001, R = 0.328), as well as between the Kelantan and Penang (p = 0.001, R = 0.380). Similarly, a separation for the Malay population between Kelantan and Penang children (p = 0.001, R = 0.249) was observed. The separation is significant by the statistical analysis of Analysis of Similarities (ANOSIM) and can also be clearly seen in the PCoA plots (Fig. 1a,c).

As for the weighted PCoA plots (Fig. 1b,d), there was a separation between the Guangzhou and Kelantan Chinese (p = 0.001, R = 0.260) but there was no separation between the Guangzhou and Penang Chinese, as well as between the Penang and Kelantan Chinese. On the other hand, there was a separation between the Kelantan and Penang Malay children at p-value of 0.001 and R-value of 0.235 (Table S1).

The results of the unweighted PCoA plots show that the types of bacteria present in the microbiota of the children population from the separate locations are different from each other, as evident from the separation for the pairwise comparisons (Table S1). On the other hand, the results of the weighted PCoA plots show that the relative abundance of some bacterial types in the microbiota is significantly different between the Guangzhou and Kelantan Chinese children, as well as between the Penang and Kelantan Malay children. However, there was no difference in the relative bacterial abundances for the other two pairs.

**Figure 2:**
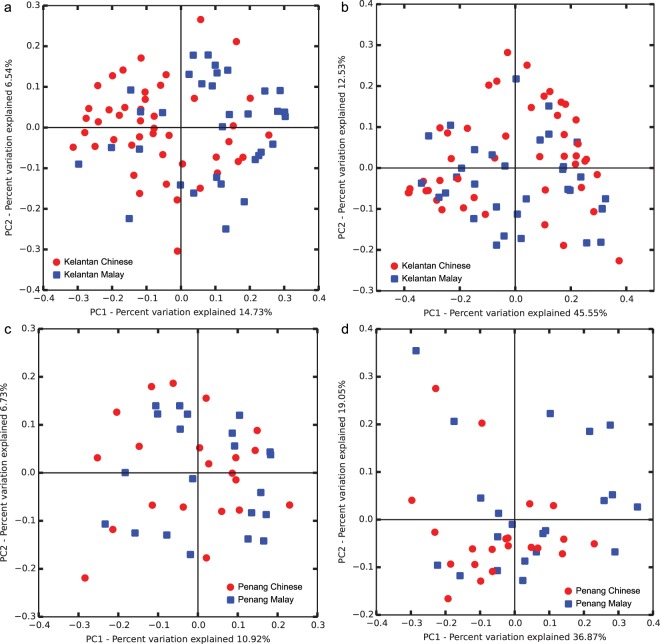
Unweighted and weighted UniFrac PCoA plots of microbiota between two ethnicities in the same location. Chinese and Malay population in two countries (Kelantan and Penang) comparison were separately plotted for race comparison in unweighted PCoA (**a**,**c**) and weighted (**b**,**d**). The statistical analysis of ANOSIM was shown in Supplementary Table S2.

The children of different ethnicities living in the same location were compared to each other (Fig. 2). For the Kelantan Malay and Chinese population, there was a separation between them in the unweighted PCoA plot (p = 0.001, R = 0.164) but no separation for the weighted PCoA plot (Fig. 2a,b and Supplementary Table S2). As for the Penang Malay and Chinese population, there was also no separation between them for both the unweighted and weighted PCoA plots (Fig. 2c,d and Table S2).

The results of the unweighted PCoA plots show that the types of bacteria present in the microbiota of Kelantan Chinese and Malay children are slightly different from each other as there was a separation in the unweighted PCoA plot. On the other hand, the microbiota of the Penang Chinese and Malay children are very similar as there was no separation between their microbiota. As for the weighted PCoA plots, the results show that the microbiota of the two different ethnicities in the same locations are very similar to each other as there was no separation for both the weighted PCoA plots.

**Figure 3:**
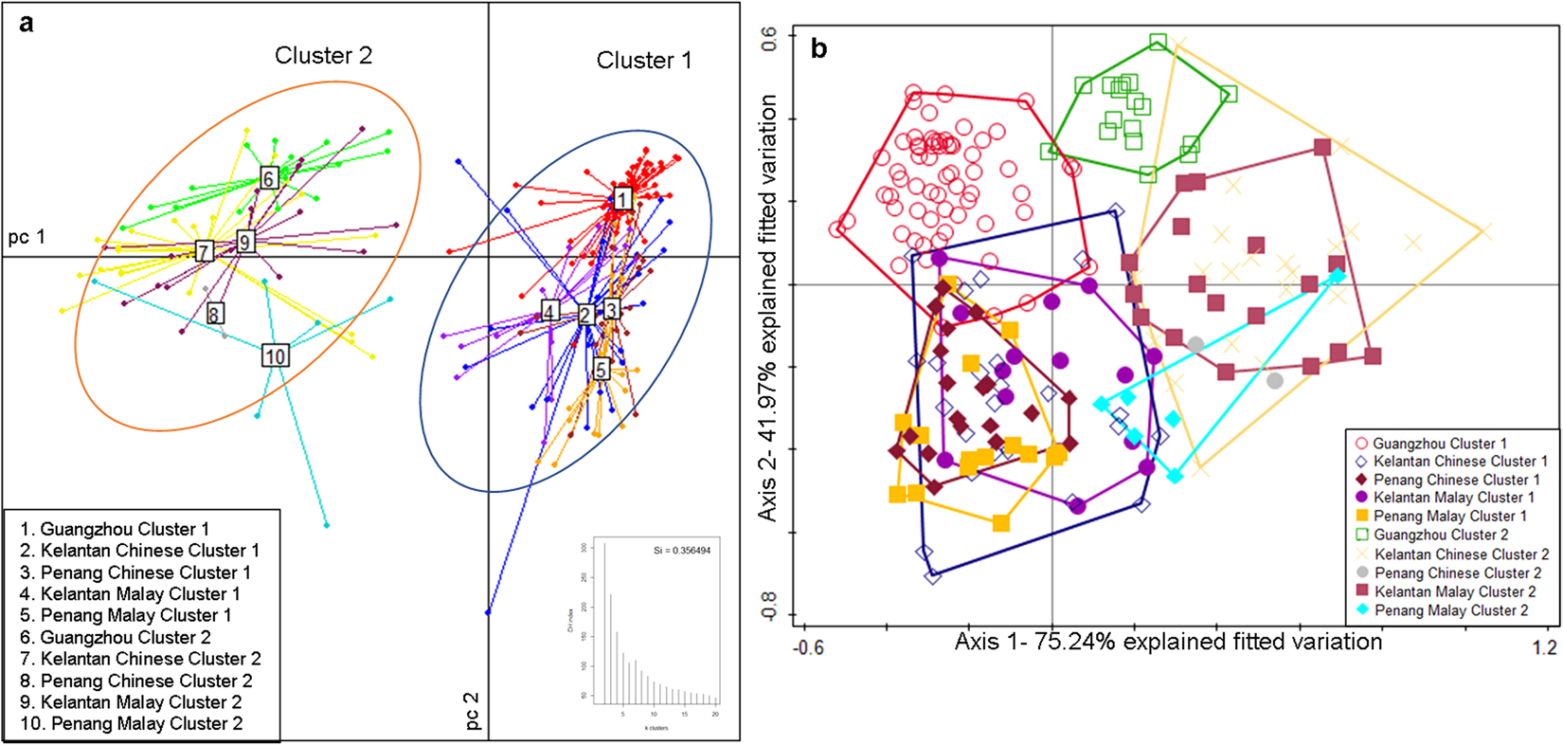
PCoA plot of PAM clustering using Jensen-Shannon distance (JSD) and db-RDA using Bray-Curtis distance comparing similarities between the two races from the three cities. Samples were revealed into two clusters clearly using PAM methods based on JSD distance matrix and bacterial genus abundances. (**a**) The two clusters were circled and within each cluster, the different races from the three cities were described in the different colors of centroid connectors. The Calinski-Harabasz (CH) index including the average silhouette (Si) coefficient was also shown. db-RDA plot was illustrated using Bray-Curtis distance and grouped into two clusters which estimated from PAM clustering method. (**b**) The percentage of variation explained for each axis is shown and the Monte Carlo test was significant at p = 0.002 and 499 permutations.

In order to confirm the separation of sample clusters by the race factor, a Partitioning Around Medoids (PAM) Clustering approach and a Distance-based Redundancy Analysis (db-RDA) involving 175 identifiable genera were performed (Fig. 3a,b). It was interesting to observe that two optimized clusters (1 and 2) were found among each race in each city validated by a mean silhouette width of 0.356 (Fig. 3a). 68.16% and 31.84% of people belong to cluster 1 and 2 respectively. The total variation observed was 13.97% and the Monte Carlo test was significant (p = 0.002) at 499 permutations (Fig. 3b). Nevertheless, separation of Guangzhou Chinese from that of Penang and Kelantan Chinese could be clearly seen within the same cluster (Fig. 3, Supplementary Tables S3 and S4), particularly in Cluster 1. Moreover, the Chinese and Malay population of Penang and Kelantan within the same cluster are closer to each other and separation is not obvious as that of Guangzhou Chinese (Fig. 3, Supplementary Tables S3 and S4). Both methods suggested that the relative abundance of the bacteria reflects the distance between the cities and the types of bacteria determine the different clusters.

**Figure 4:**
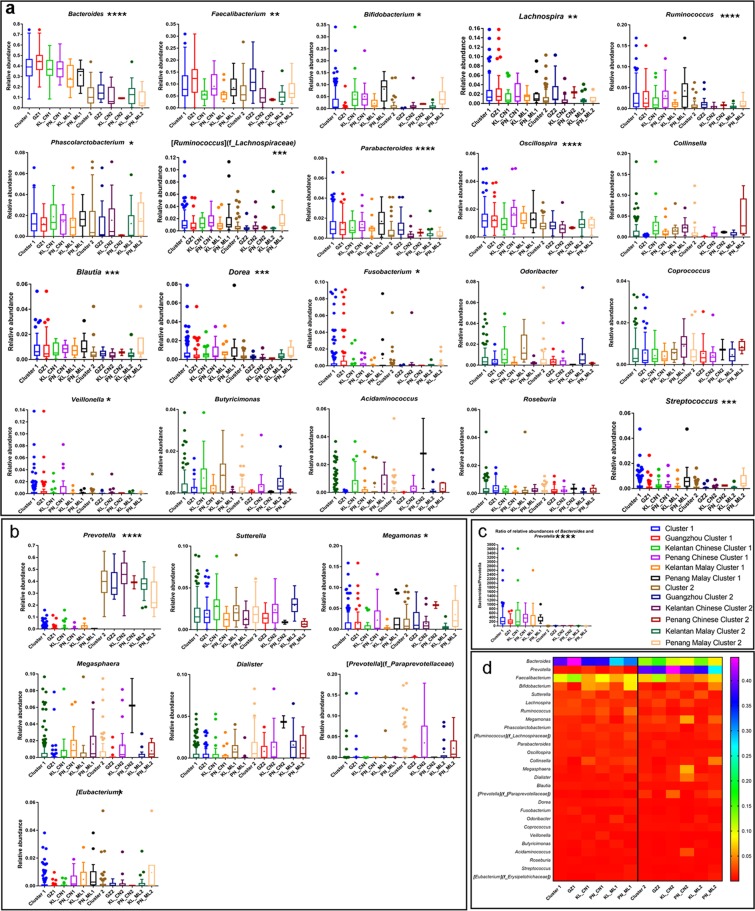
Relative abundance of major bacteria among the two ethnicities and the three different cities. Individual top 1% of the bacterial genera were plotted as box-plots. 20 bacteria were abundant in Cluster 1 (**a**) and 7 bacteria were rich in Cluster 2 (**b**). The ratio of Bacteroides and Prevotella abundances were compared (**c**). The symbol keys will be represented for (**a**–**c**) Overall major bacteria profile was shown as a heatmap (**d**). Relative abundances of OTU were illustrated as a spectrum of colors. The significant bacteria were marked with asterisks in a, b and c. Two-tailed ****p < 0.0001, ***p ≥ 0.0001–<0.001, **p ≥ 0.001–<0.01, *p < 0.05.

In comparing Operational Taxonomy Units (OTUs) above 1% of the total population of bacterial genera, total 17 bacterial genera were found significantly different between cluster 1 and 2 using the Mann-Whitney U test (Fig. 4 and Supplementary Table S5). Cluster 1 was dominated by 14 bacteria (Fig. 4a) and *Bacteroides* was the most abundant, whereas Cluster 2 was dominated by 3 bacteria and led by *Prevotella* (Fig. 4b). The ratio of both bacteria was found comparable within each cluster, but significantly different between two clusters across the different ethnicities and cities (Fig. 4c). Those major bacteria were found in the abundances order in a heatmap (d). *Bacteroides*, *Prevotella*, *Fecalibacterium*, and *Bifidobacterium* were the top 4 bacteria and the respective significant bacteria in pairs were found (Supplementary Tables S6–S8).

The predominant bacteria, with abundances above 1% of the total bacterial population in the two clusters are shown in Fig. 4d. Kruskal Wallis statistical test followed by Dunn’s post-hoc multiple comparison tests were performed (Supplementary Tables S6–S8). In a pairwise comparison of each of the bacterium between the two clusters, in the same city and race, the abundance of *Bacteroides*, *Prevotella*, *Parabacteroides*, and *Streptococcus* was significantly different and may have contributed to the differentiation of the clusters (Supplementary Table S6). *Bacteroides, Parabacteroides*, and *Streptococcus* were predominant members of Cluster 1, whereas, *Prevotella* was the most abundant in Cluster 2.

Within the same city, the abundant of the four predominant bacteria (*Bacteroides, Prevotella, Oscillospira, Dorea, and Odoribacter*) were found to be significantly different between the two ethnicities (Supplementary Table S7). However, between the same ethnicity in the three different cities, numerous bacteria (18 out of the 27 of those above 1% of bacterial population) showed differences in their abundance, which suggests that geographical location-associated factor(s) but not the ethnicity is the major determinant of the bacterial abundance (Supplementary Table S8).

### Effect of diet on microbiota

**Figure 5:**
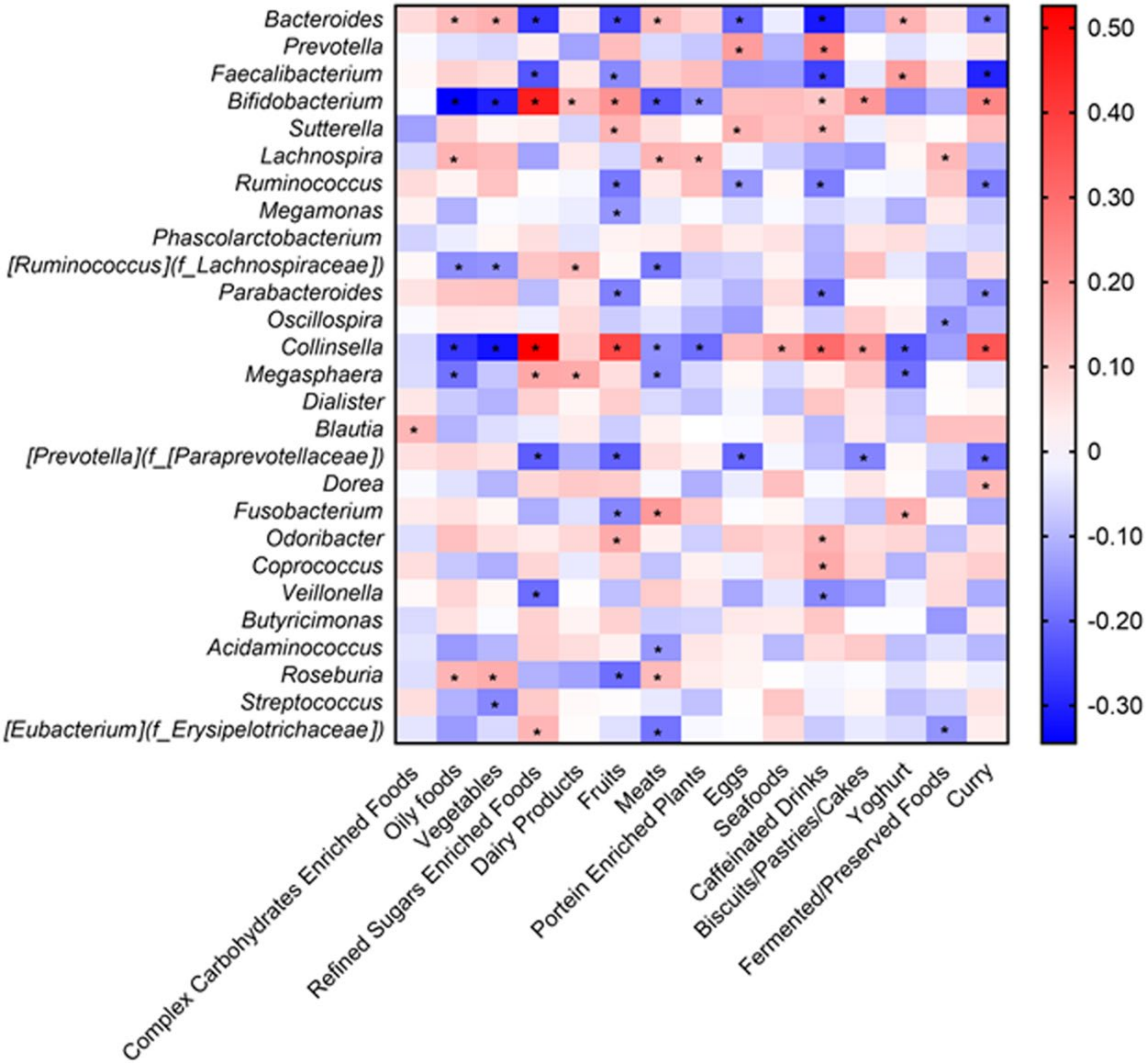
Correlation between the quantity of the various food types consumed in the fraction and bacterial abundances at the genus level. The food types and bacterial abundances were described in different axes. The Spearman rho (r) values were reflected as a color gradient and positively and negatively correlations are found in red and blue spectrums. The significant correlation is shown as asterisks * two-tailed p < 0.05.

As diet could be a geolocation-associated causative factor in influencing gut microbiota, the effects of the diet were investigated. The correlation between the portion of 15 food types (complex carbohydrates enriched foods, oily foods, vegetables, refined sugars enriched foods, dairy products, fruits, meats, protein enriched plants, eggs, seafood, caffeinated drinks, biscuits/pastries/cakes, yoghurt, preserved foods and curry) and bacterial abundances at the genus level is shown in Fig. 5.

Out of the 27 bacteria compared, while there are many significant correlations, there are four genera that stood out with high rho values (≥±0.3) among the 15 food types. The four genera are *Bacteroides, Fecalibacterium, Bifidobacterium*, and *Collinsella*. (Supplementary Table S9). Interestingly, all correlated with oily foods, vegetables, refined sugars enriched foods, fruits, meats, and curry and caffeinated drinks. Looking into the correlation between bacteria and the foods, *Bifidobacterium* and *Collinsella* are positively correlated with refined sugars enriched foods, while the latter is also positively correlated with fruits and curry foods. However, *Bacteroides*, *Faecalibacterium*, *Bifidobacterium*, and *Collinsella* are negatively correlated with caffeinated drinks, curry, oily foods, and Southeast Asian vegetables respectively.

## Discussion

The results show one main point, which is that the difference in microbiota composition is mainly due to geographical location, be it locational dietary habit, ethnicity or environmental influence. When compared the different ethnicities living in the same location, there was no significant difference in the microbiota composition within the same cluster according to both PAM Clustering and dbRDA analysis. On the other hand, when children of same ethnicity living in separate locations, in particular, Chinese in Guangzhou and the two Malaysian cities within Cluster 1 were compared, there was a significant difference in the microbiota composition and abundance according to dbRDA analysis. Thus, the microbiota profile is not to any significant extent determined by ethnicity among the children. In a previous study on dichorionic triplet sets have shown that genetics do affect gut microbiota differences, but only at one month of age and after which, environmental factors play a larger role^17^.

It is reasonable to assume that the subjects of separate locations are being exposed to different environments or lifestyles (cultures) and have access to different food types and hence will have different microbiota. A recent study done in mice has shown that mice in individually ventilated cages have different microbiota as compared to mice who live together in the same cage when they were all initially implanted with the same gut microbiota^18^. It was also shown that the microbiome similarity was found among the genetically unrelated individuals who shared household items^14^. This suggests that the environment can affect gut microbiota and those in the same environment will have similar gut microbiota types.

However, in our study among the children, microbiota profiles of an ethnicity (e.g. Chinese) resided in the same city (e.g. Guangzhou or Kelantan) are differentiated into the two clusters; while the same ethnicity in the same cluster (e.g. Chinese or Malay) living in distant cities (e.g. Penang and Kelantan) with different living environment and lifestyle (Penang being urban, while the Kelantan is rural) have similar microbiota profiles. This strongly suggested microbiota profile is to a little extent influenced by the living environment and further confirmed that ethnicity is not the major determining factor.

Despite different ethnicities, their food culture might have become more similar for the children in the same city (both Cluster 1 and 2). Malaysian cities are populated by Chinese and Malay, and thus most people consumed both Chinese and Malay types and fusion foods. Hence, it is no surprise that the microbiota of the Malaysian Chinese and Malay are very similar. On the other hand, the preferred food type is a personal choice and may explain the segregation in the microbiota between the clusters even within a city.

Among the three cities, higher consumption of refined sugar enriched foods, fruit, and curry led to a higher abundance of *Bifidobacterium* and *Collinsella* (r ≥±0.3); whereas higher consumption of curry led to a higher abundance of *Faecalibacterium* and *Collinsella*. On the other hand, oily food and vegetable consumption was negatively associated with *Bifidobacterium* and *Collinsella* respectively. *Bacteroides, Faecalibacterium* and *Bifidobacterium* are the predominant bacteria in Cluster 1. The relative proportion of refined sugar, fruit, curry, oily food and vegetable, and perhaps not a single food item in the diet may have determined Cluster 1 profile. High fat consumption has been reported to associate with *Bacteroides*^7,19–21^. Cluster 2 is dominated by *Prevotella* and was not found to be strongly associated, positively or negatively with any of the food types in this study. Dietary fiber has been reported to promote *Prevotella* abundnace^7^. Nevertheless, *Prevotell*a is negatively associated with *Bacteroides*, as observed in this study and others^7,19–21^.

A study done on Malaysian children of three ethnic groups namely Chinese, Malay, and aboriginal Orang Asli showed that the Chinese and Malay children share greater bacterial genetic lineages then when compared to the Orang Asli^22^. While one of the reasons could be the difference in dietary habit due to socioeconomic status, another reason they suggested was that the Chinese and Malay children were exposed to similar food types^22^. This agrees with the results of this study that diet is the main determinant of gut microbiota.

Interestingly, the predominant microbiota detected among Southern Han Chinese (Guangzhou in this study), are different from those found among the central and Northern Han Chinese (Wuxi, Chengdu, Zhengzhou and Harbin), where the gut microbiota of the youngsters was largely populated by *Phascolarctobacterium, Roseburia, Bacteroides, Blaustia, Faecalibacterium* and *Clostridium* in that order^23^. The gut microbiota profile of Guangzhou children is different from that of the central and Northern Chinese, but closer to that of the Southeast Asian.

Another interesting observation is that the Guangzhou children have almost no *Bifidobacterium* in their microbiota, as it is inversely proportional to the abundance of *Bacteroides*. This is of concern as many of the *Bifidobacterium* species are known to have health benefits. While *B. adolescentis* have been used for manufacturing of functional dairy products, *B. longum* lowers the risk of diarrhea and allergies in infants^24,25^, and several other *Bifidobacterium* species show benefits to gastrointestinal health as well. Hence, it would be relevant to investigate the gut microbiota health of Guangzhou children to verify if the lack of *Bifidobacterium* indeed resulted in a higher occurrence of gut-related diseases.

Diet is a stronger influencing factor that can affect the changes in the relative abundance of gut microbes. From the correlation tables, it can be seen that the consumption of one food type can correlate with many genera and a single genus can also correlate with many food types. This shows that the food consumed is not exclusively used by one type of bacteria and the bacteria also do not exclusively feed on one type of food. The correlation between diet and microbiota is multidimensional, future metatranscriptomic and metabolomic studies would provide further understanding in their complex interrelation and may allow better modulation of microbiota through dietary intervention.

## Conclusion

In summary, differences in the gut microbiota are largely due to dietary habit. Living environmental condition, and ethnicity are not determining factors for Southern Chinese and Southeast Asian children.

## Methods

### Ethics approval and consent to participate

The respective Institutional Review Board (IRB) at Guangzhou, Penang, Kelantan universities reviewed and approved the informed consent and the study protocols and other documents. The Joint Ethics Committee on Clinical Studies of School of Pharmaceutical Sciences, Universiti Sains Malaysia and Hospital Lam Wah Ee (JEC-SPS, USM & HLWE) (USM-HLWE/IEC/2014-0006) confirmed that the study was in compliance with the declaration of Helsinki and Malaysia Good Clinical Practice guidelines.

### Study Design

A total of 201 local-born healthy children between 7 and 12 years old of both genders (male n = 110, female n = 91) were recruited at the respective schools from three cities: Guangzhou Southern Han Chinese (n = 81), Penang Southern Han Chinese (n = 21), Penang Malay (n = 21), Kelantan Southern Han Chinese (n = 45) and Kelantan Malay (n = 33). The participants who were healthy 7–12 years old children should not consume any pre/pro-biotics and antibiotics 2 weeks and 3 weeks prior respectively and have any recent illness that required to get medical attention last 3 months. All the participants had been informed and the consented documents were received from a parent upon the collection of samples.

### Dietary intake questionnaires

Subjects were given the food frequency questionnaire (FFQ) requesting them to fill in the number of times they consume a specific type of food on a daily, weekly or monthly basis. We adopted the validated FFQ developed by the Singapore Health Promotion Board. The complete FFQs (Supplementary Text S12) and the raw data can be found. The questionnaire focuses on types, frequency, and the portion of foods and ingredients being consumed. We characterized the food items into 15 food types such as complex carbohydrates enriched foods, oily foods, vegetables, refined sugars enriched foods, dairy products, fruits, meats, protein enriched plants, eggs, seafood, caffeinated drinks, biscuits/pastries/cakes, yoghurt, preserved foods, and curry.

### Collection of samples and DNA extraction

Approximately 10 g of stool was collected in 2 ml of RNA*later*® (Ambion, Inc., Texas, USA) and 0.2 ml of fecal homogenate was further washed with 1x Phosphate-buffered saline (PBS). The DNA extraction was done using the TIANamp Stool DNA kit (TIANGEN Biotech, Co., Ltd., Beijing, China) according to the manufacturer’s protocol. Detail protocol (Supplementary Text 10) could be accessed.

### Next-generation Sequencing

Once the concentration of the extracted double-stranded DNA had been measured using the Quant-iT^TM^ Picrogreen® kit (Invitrogen, Inc., Carlsbad, USA), it was amplified with a Nextera® transposase sequences (details at S11 Text in supplementary information) and KAPA HiFi^TM^ PCR kit (Roche life science, Inc., Indiana, USA). The PCR products were then purified by Agencourt® AMPure XP beads (Beckman Coulter, Inc., Fullerton, USA). In the index PCR, the reaction mixture was attached with the Illumina indexes (Illumina, Inc., San Diego, USA) using the KAPA HiFi^TM^ PCR kit The DNA libraries produced were purified again using the Agencourt® AMPure XP beads. After the quantity of each library had been checked, it was normalised and pooled as Pooled Amplicon Library (PAL). Then, the PAL was then re-quantified with qPCR using the KAPA Library Quantification Kit (Roche life science, Inc., Indiana, USA).

The PAL and the internal control library (Illumina, Inc., San Diego, USA) were denatured with sodium hydroxide (NaOH) and diluted with a pre-chilled hybridization buffer (HT1) to the appropriate final concentration. The solution was sequenced on the Illumina® Miseq Desktop Sequencer (Illumina, Inc., San Diego, USA) The detailed protocol of the 16S rRNA sequencing preparation was described in Supplementary Text 11.

### Data analysis

#### Bioinformatics analysis

The sequence data obtained were analyzed using QIIME (Quantitative Insights Into Microbial Ecology) version 1.9.1^26^. Using the QIIME the forward and reverse reads of the same sample were first joined. The paired reads were demultiplexed and quality filtered at Q-score of 25. Chimeric sequences were filtered out and Non-chimera were selected by USEARCH v6.1^27^. After which, using Greengenes v13.8 reference database, open-reference operational taxonomic units (OTUs) were picked out from the non-chimeric sequences at 97% similarity. The OTUs were summarized into taxonomy profiles of the bacteria in each sample. The bacterial genus data was provided. Beta diversity was calculated using the UniFrac distance matrices, generating the two- dimensional Principal Coordinate Analysis plots (PCoA). Weighted and unweighted UniFrac distance matrices were used to derive the beta diversity of the samples^28^ which data could be accessed.

The relative abundance of genus data was filtered because the unassigned fraction was not considered in the calculation of clustering, distance-based redundancy analysis (db-RDA), the taxonomy classification and their statistical analysis. In total 175 genera were identified. A constrained distance-based redundancy analysis (db-RDA) was performed with the software CANOCO 5 (Microcomputer Power, USA). A relative abundance of bacterial genus level OTU data was applied and a classified triplot was drawn using Bray Curtis distance according to the manufacturer’s instructions^29^.

### Clustering

Applying the approach of Enterotyping^30,31^ using R version 3.5.1, the Jensen-Shannon distance (JSD) matrix was calculated based on the 175 relative abundances. The matrix was clustered by the Partitioning Around Medoids (PAM) algorithm using cluster package. Calinski-Harabasz (CH) index allowed estimation of the optimal number of clusters. The cluster number was validated using the individual and average silhouette coefficient (Si) and visualized by PCoA plots using the JSD matrix and adegraphics R package.

### Statistical analysis

ANOSIM (Analysis of similarities) in QIIME was used to test for degree of separation between groups compared by using beta diversity Unifrac distance matrices as input. R-value and p-value shown in Table S1 and Table S2 were derived from Anosim test on the pairwise comparison based on unweighted and weighted Unifrac distance matrices. P-values < 0.01 are indicated with a * and shows significant separation.

For the bacteria at the genus level, only the top 1% highest abundance for each population were used for statistical analysis by GraphPad Prism 7 (GraphPad Software Inc., San Diego, USA). The non-parametric ANOVA; Kruskal-Wallis test, Mann-Whitney U test, and Multiple comparison post-hoc Dunn’s test were performed to check for significant differences in the distribution of bacteria between the groups compared.

As for the diet data, the types of food consumed were classified into 15 different groups according to the FFQs, based on the frequency and portion of the food types consumed per month for every subject, and expressed as a fraction of total foods consumed. In addition, the bivariate Spearman rank correlation was done for correlation between the abundance of bacteria present and consumption of a food type.

A Permutational multivariate analysis of variance (PERMANOVA) test was performed for the JSD and the Bray Curtis distance matrices using pairwiseAdonis (pairwise comparison of vegan R package) followed by Bonferroni post-hoc test. For db-RDA model, Monte Carlo permutation test with 499 random permutations was also performed and pseudo F values, pseudo F statistics, and p values were generated.

## Supplementary information

Supplementary Tables S1, S2, S3, S4, S5, S6, S7, S8, S9

Supplementary Texts S10, S11, S12

## Data Availability

The dataset generated or analysed during this study are available in the KNB repository, doi:10.5063/F12B8W9B and the rest of the data and materials are provided as the Supplementary Information Files.
