# Supplementary material for: Gut microbiome of pre-adolescent children of two ethnicities residing in three distant cities
Source: Sci Rep. 2019 May 24;9:7831. doi: 10.1038/s41598-019-44369-y (PMC6534553; doi:10.1038/s41598-019-44369-y)

**Supplementary materials**

Gut microbiome of pre-adolescent children of two ethnicities residing in three distant cities

Wei W.T. Khine^1,2^, Yuwei Zhang^1^, Gerald J.Y Goie^1^, Mung Seong Wong^3^, Mintze Liong^4^, Yeong Yeh Lee^3^, Hong Cao^5^, Yuan-Kun Lee^1,6*^

**Supplementary Text S10: Fecal Bacterial DNA extraction protocol**

**Materials**

TIANamp Stool DNA kit (Tiangen, #DP328)

**Steps**

1. Pipet 0.2 ml of stool homogenate into the microcentrifuge tube and mix with 1 ml of 1x PBS.
2. Centrifuge at 13,000 rpm for 1 minute and discard the supernatant.
3. Repeat once and continue the following steps with the fecal pellet.
4. Add 1.4 ml GSL buffer to each fecal pellet. Vortex continuously for 1 min or until it is thoroughly homogenized.
5. Heat the suspension for 5 min at 95°̇C. Vortex the suspension for 15 sec then, centrifuge at 13,000 rpm for 1 min. Transfer 1.2 ml of supernatant to a new 2 ml microcentrifuge tube.
6. Add 1 InhibitEX tablet to each sample and vortex continuously for 1 min or until it is thoroughly suspended.
7. Incubate the suspension for 1 min at room temperature. Centrifuge at 13,000 rpm for 3 min. Transfer all supernatant to a new 2 ml microcentrifuge tube.
8. Add 0.015 ml Proteinase K. Add 0.2 ml GB buffer and vortex for 15 sec.
9. Incubate at 70̇°C for 10 min and centrifuge briefly.
10. Add 0.2 ml ethanol (96- 100%) to the sample and mix thoroughly by vortexing for 15 sec. Centrifuge briefly.
11. Pipet all the mixture into TIANamp spin column CR2 and centrifuge at 13,000 rpm for 30 sec. Discard the flow-through and place the spin column into the collection tube.
12. Add 0.5 ml GD buffer to TIANamp spin column CR2 and centrifuge at 13,000 rpm for 30 sec. Discard the flow-through and place the spin column into the collection tube.
13. Add 0.7 ml PW buffer to TIANamp spin column CR2 and centrifuge at 13,000 rpm for 30 sec. Discard the flow-through and place the spin column into the collection tube.
14. Add 0.5 ml PW buffer to TIANamp spin column CR2 and centrifuge at 13,000 rpm for 30 sec. Discard the flow-through and place the spin column into the collection tube.
15. Centrifuge at 13,000 rpm for 2 min to dry the membrane completely. Place the TIANamp spin column CR2 in a new sterile 2 ml microcentrifgure tube.
16. Add 0.05 ml TE buffer directly to the center of the membrane. Incubate at room temperature for 7 min and centrifuge at 13,000 rpm for 2 min.
17. Check the concentration and purity (A260/280) of the eluted DNA with Nanodrop and store at -20°C.

**Supplementary Text S11: 16S rRNA Metagenomic sequencing library preparation protocol**

Metagenomic studies are commonly performed by analyzing the prokaryotic 16S ribosomal RNA (16S rRNA) gene. This protocol describes a workflow for preparing samples for sequencing the V3-V4 regions of the 16S rRNA gene.

**Materials**

1. KAPA HIFI HotStart ReadyMix (Kapa Biosystems, #KK2602)

2. 16S Amplicon PCR Forward Primer-5 (Sigma Aldrich) TCGTCGGCAGCGTCAGATGTGTATAAGAGACAGCCTACGGGNGGCWGCAG

3. 16S Amplicon PCR Reverse Primer-5 (Sigma Aldrich) GTCTCGTGGGCTCGGAGATGTGTATAAGAGACAGGACTACHVGGGTATCTAATCC

4. AMPure XP beads (Beckman Coulter, #A63881)

5. Nextera XT Index kit (Illumina, #FC-131-1002)

6. Quanti-iT PicoGreen dsDNA reagent and kits (Life Technologies, #P7589)

7. KAPA library quantification kit (Kapa Biosystems, #KK4824)

8. Miseq Reagent kit V2 500 cycles (Illumina, #MS102-2003)

**Steps**

1. PCR Amplification

1. Dilute each extracted DNA sample to a concentration of 12.5 ng.
2. Make up the following reagent / composition.

Composition of PCR reagents for 16S amplicon generation.

Set up the following reaction of DNA, 2x KAPA HiFi HotStart ReadyMix, and primers:

|  | Volume |
| --- | --- |
| Microbial DNA (5 ng/µl) | 2.5 µl |
| Amplicon PCR Forward Primer 10 µM | 0.5 µl |
| Amplicon PCR Reverse Primer 10 µM | 0.5 µl |
| 2x KAPA HiFi HotStart ReadyMix | 12.5 µl |
| Total | 25 μl |

1. Put on thermocycler for the following conditions:

Initial denaturation - 95°C for 3 mins

Denaturation - 95°C for 30 sec

25 cycles

Annealing - 55°C for 30 sec

Extension - 72°C for 30 sec

Final extension - 72°C for 5 min

Hold at 4°C

2. PCR Cleanup 1

- Using a multichannel pipette, add 20 µl of AMPure XP beads to each well of the Amplicon PCR plate.
- Incubate PCR product and magnetic bead mixture at room temperature for 5 minutes.
- With the Amplicon PCR plate on the magnetic stand, use a multichannel pipette to carefully remove and discard the supernatant.
- With the Amplicon PCR plate on the magnetic stand, wash the beads with freshly prepared 80% ethanol as follows:
- Using a multichannel pipette, add 200 µl of freshly prepared 80% ethanol to each sample well.
- Incubate the plate on the magnetic stand for 30 seconds.
- Carefully remove and discard the supernatant.
- Repeat the 80% ethanol washing step.
- With the Amplicon PCR plate still on the magnetic stand, allow the beads to air‐dry.
- Remove the Amplicon PCR plate from the magnetic stand. Using a multichannel pipette, add 52.5 µl of 10 mM Tris pH 8.5 to each well of the Amplicon PCR plate.
- Mix and incubate at room temperature for 2 minutes.
- After the supernatant is clear, transfer 50 µl of the supernatant to new plate.

3. PCR Amplification

- Set up the following reaction of DNA, Index 1 and 2 primers, 2x KAPA HiFi HotStart Ready Mix, and PCR Grade water:

|  | **Volume** |
| --- | --- |
| DNA | 5 µl |
| Nextera XT Index Primer 1 | 5 µl |
| Nextera XT Index Primer 2 | 5 µl |
| 2x KAPA HiFi HotStart Ready Mix | 25 µl |
| PCR Grade water | 10 µl |
| Total | 50 μl |

- Perform PCR on a thermal cycler using the following program:
- 95°C for 3 minutes
  - 95°C for 30 seconds

8 cycles

- - 55°C for 30 seconds
  - 72°C for 30 seconds
- 72°C for 5 minutes
- Hold at 4°C

# 4. PCR Clean‐Up 2

- Perform above workflow section 2 using 56 µl of AMPure XP beads and 27.5 µl of 10 mM Tris pH 8.5.
- Elute 25 µl of supernatant from index PCR plate to a new 96-well PCR plate.

# 5. Library Quantification, Normalization, and Pooling

- Check DNA concentration in nM, based on the size of DNA amplicons as determined by using fluorescent nucleic acid stain

(Concentration in ng/µl) x 10^6^ = concentration in nM

(660 g/mol x average library size)

- Dilute concentrated the final library using 10 mM Tris pH 8.5 to 4 nM.
- Aliquot 5 µl of diluted DNA from each library and mix aliquots for pooling libraries.

6. Pooled library re-quantification by qPCR

- Dilute libraries template as accordingly.
- Prepare reaction mix

1. Prepare the qPCR master mix as follows:

| Consumable | ul / well |
| --- | --- |
| KAPA SYBR qPCR MasterMix with primer | 12 |
| PCR grade water | 4 |
| DNA Standards or diluted library | 4 |
| Total | 20 |

- Place the 96-well plate in the qPCR machine and set-up the following thermal profile:

| **Procedure** | **Temperature (°C)** | **Time** |
| --- | --- | --- |
| KAPA Hotstat  x 40 | 95 | 3 mins |
|  | 95 | 20 sec |
|  | 60 | 30 sec |

- Calculate the initial concentration of your unknown library templates based on the standard curve generated from the control template dilutions.

Library concentration (nM) =

Measured conc (pM) x dilution factor

1000

Standard amplicon size

Library amplicon size

x

# 7. Library Denaturing and MiSeq Sample Loading

### (A) Denature DNA

- Calculation of pooled library concentration will be changed according to qPCR result. Following concentrations are examples for calculation.
- Combine pooled final DNA library and freshly diluted 0.2 N NaOH in 1:1 dilution.
- Vortex briefly to mix the sample solution, and then centrifuge the sample solution at 280

× g at 20°C for 1 minute.

- Incubate for 5 minutes at room temperature.
- Add pre-chilled HT1 to the tube containing denatured DNA making 1:100 dilution till 20 pM.

### Dilute Denatured DNA

- Dilute the denatured DNA to the desired concentration using the following example:

| **Final Concentration** | **2 pM** | **4 pM** | **6 pM** | **8 pM** | **10 pM** |
| --- | --- | --- | --- | --- | --- |
| 20 pM denatured library | 60 µl | 120 µl | 180 µl | 240 µl | 300 µl |
| Pre‐chilled HT1 | 540 µl | 480 µl | 420 µl | 360 µl | 300 µl |

- Invert several times to mix and then pulse centrifuge the DNA solution.

### (B) Denature and Dilution of PhiX Control

Use the following instructions to denature and dilute the 10 nM PhiX library to the same loading concentration as the Amplicon library. The final library mixture must contain at least 5% PhiX.

- Dilute the PhiX library with 0.2 N NaOH to 4 nM from 10 nM using 10 mM Tris pH 8.5.
- Denature the diluted library with 0.2 N NaOH in 1:1 dilution.
- Vortex briefly to mix and incubate for 5 minutes at room temperature to denature the PhiX library into single strands.
- Add the pre‐chilled HT1 to the tube containing denatured PhiX library resulting in a 20 pM PhiX library.
- Dilute the denatured 20 pM PhiX library to the same loading concentration as the Amplicon library as follows:

| **Final Concentration** | **2 pM** | **4 pM** | **6 pM** | **8 pM** | **12.5 pM** |
| --- | --- | --- | --- | --- | --- |
| 20 pM denatured library | 60 µl | 120 µl | 180 µl | 240 µl | 375 µl |
| Pre‐chilled HT1 | 540 µl | 480 µl | 420 µl | 360 µl | 225 µl |

- Invert several times to mix and then pulse centrifuge the DNA solution.

### (C) Combine Amplicon Library and PhiX Control

- Combine followings:
  - Denatured and diluted PhiX control (20%)
  - Denatured and diluted amplicon library (80%)
- Using a heat block, incubate the combined library and PhiX control tube at 96°C for 2 minutes.
- After the incubation, invert the tube 1–2 times to mix and immediately place in the ice-water bath for 5 minutes.

8. MiSeq run

1. Load libraries into cartridge

Load sample in highlighted position on reagent cartridge.

1. Set up the sequencing run with Metagenomics function

**Supplementary Text S12: Food Frequency Questionnaire**


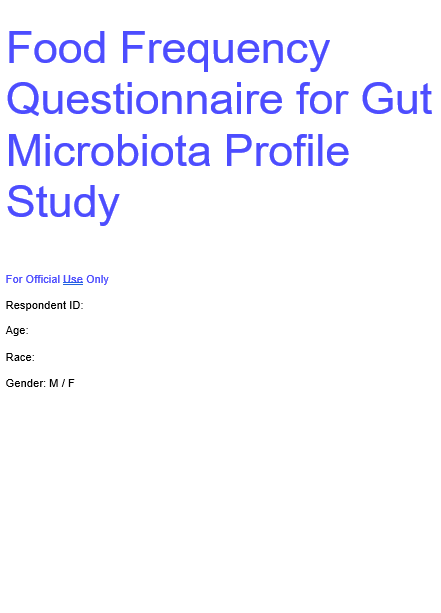


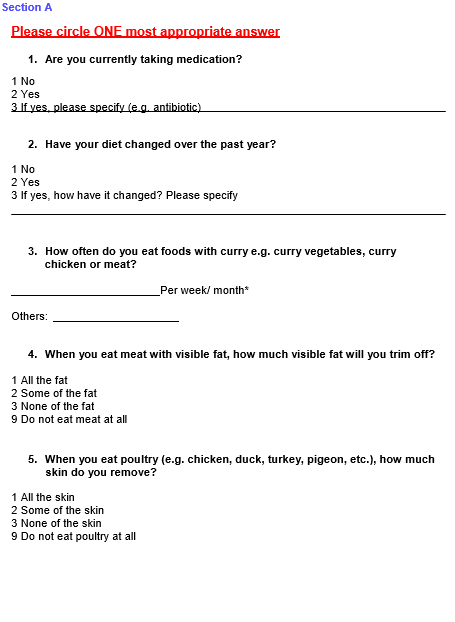


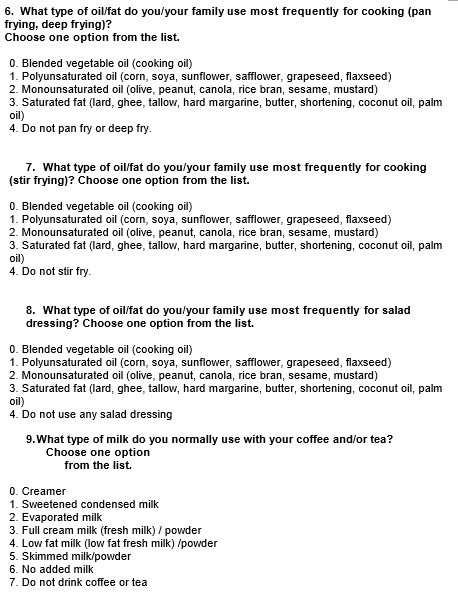


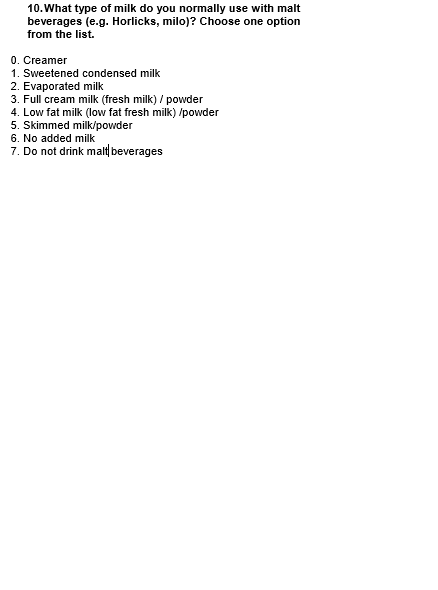


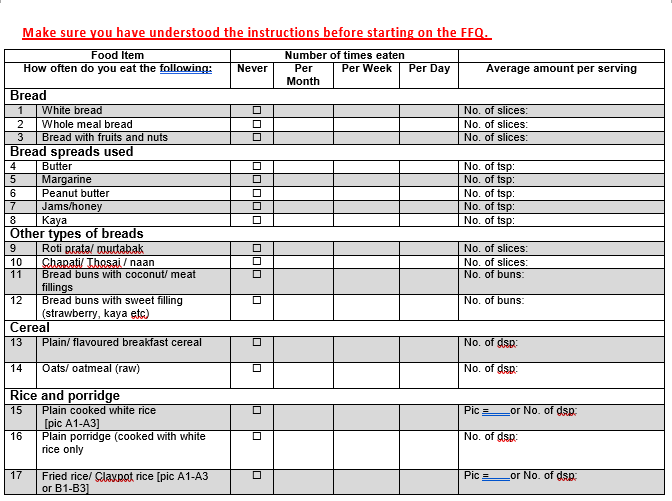


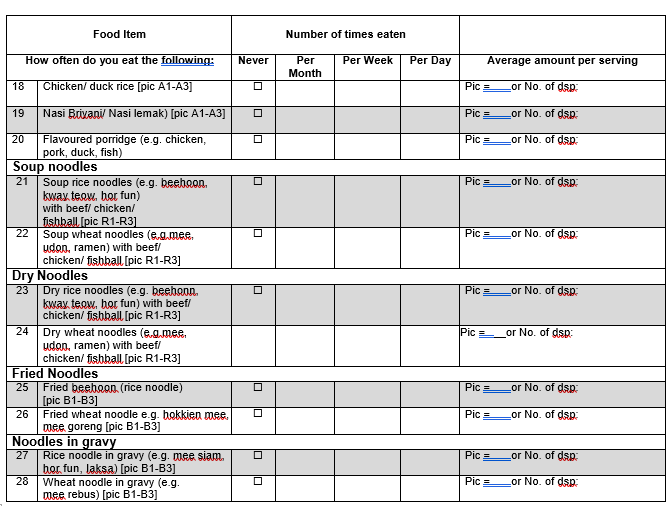


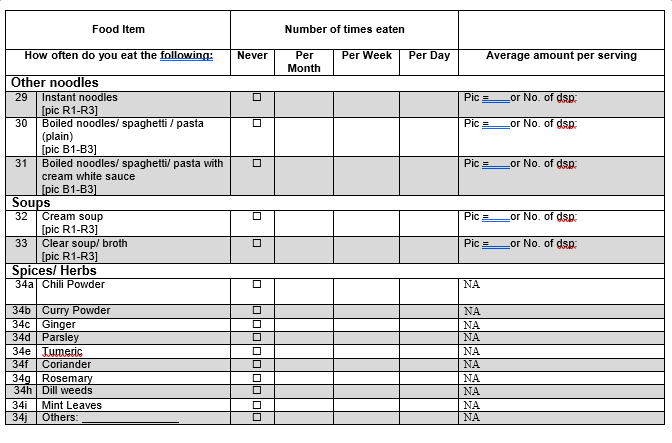


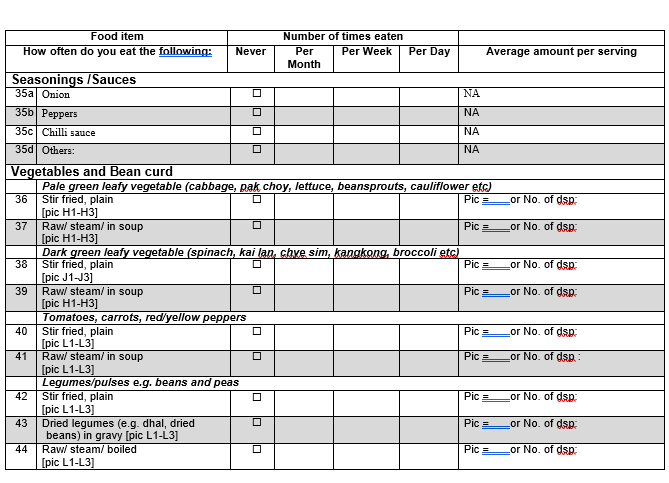


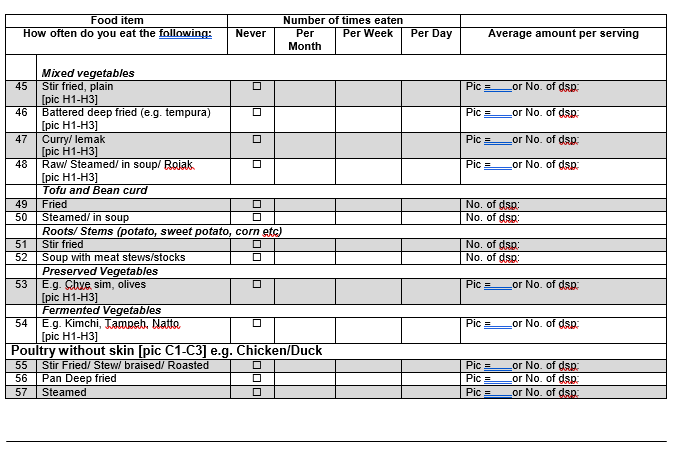


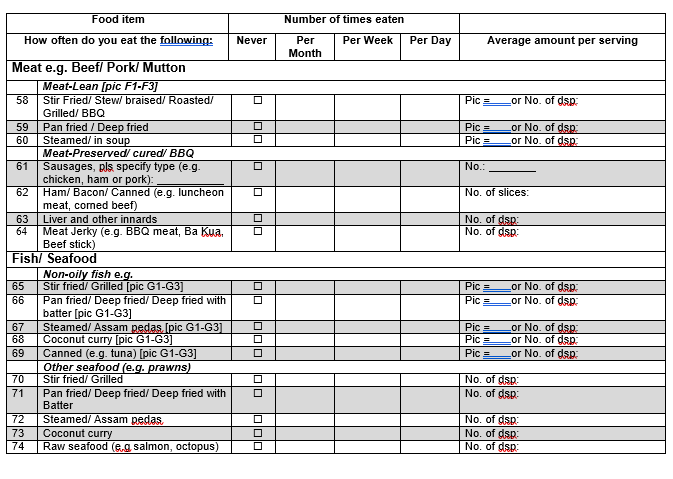

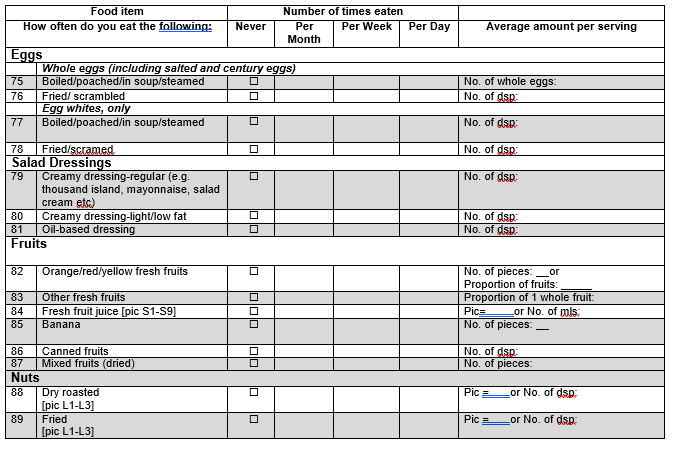


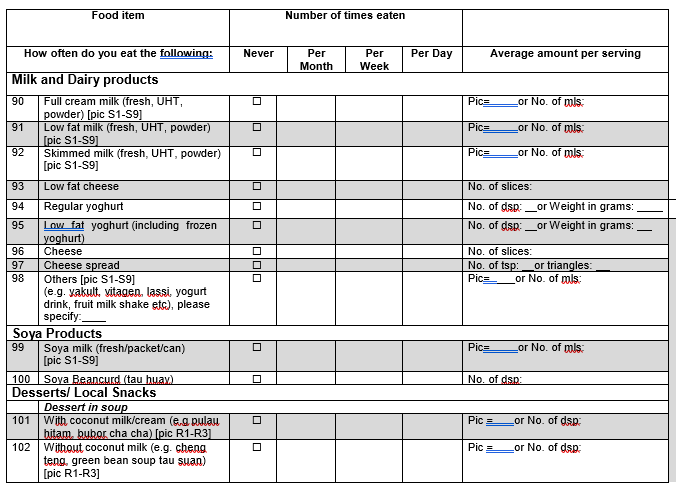


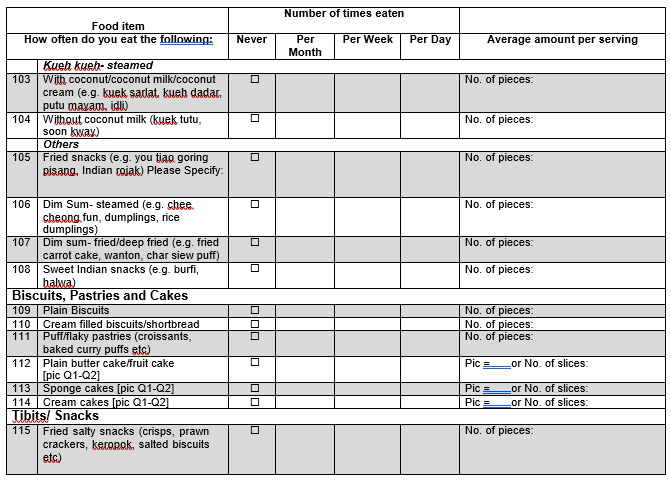


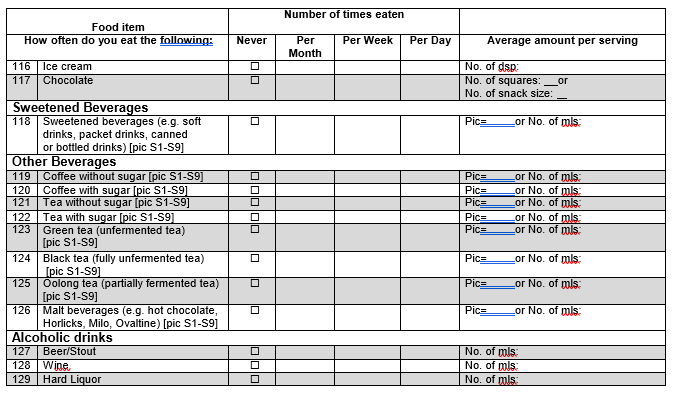


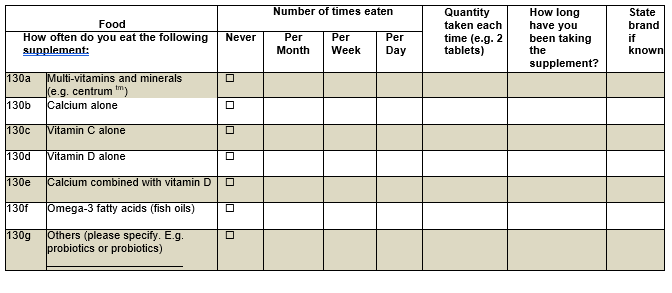

Supplement: Supplementary file 2 — Supplementary Texts S10, S11, S12 [file 41598_2019_44369_MOESM2_ESM.docx]
